# Multipolar pacing by cardiac resynchronization therapy with a defibrillators treatment in type 2 diabetes mellitus failing heart patients: impact on responders rate, and clinical outcomes

**DOI:** 10.1186/s12933-017-0554-2

**Published:** 2017-06-09

**Authors:** Celestino Sardu, Michelangela Barbieri, Matteo Santamaria, Valerio Giordano, Cosimo Sacra, Pasquale Paolisso, Alessandro Spirito, Raffaele Marfella, Giuseppe Paolisso, Maria Rosaria Rizzo

**Affiliations:** 1 Department of Medical, Surgical, Neurological, Metabolic and Aging Sciences, University of Campania “Luigi Vanvitelli”, Piazza Miraglia, 2, 80138 Naples, Italy; 2 Cardiovascular and Arrhythmias Department, John Paul II Research and Care Foundation, Campobasso, Italy; 3 Cardiovascular Department, Inselspital of Bern University, Bern, Switzerland

## Abstract

**Background:**

Type 2 diabetes mellitus (T2DM) is a multi factorial disease, affecting clinical outcomes in failing heart patients treated by cardiac resynchronization therapy with a defibrillator (CRT-d).

**Methods:**

One hundred and ninety-five T2DM patients received a CRT-d treatment. Randomly the study population received a CRT-d via multipolar left ventricle (LV) lead pacing (n 99, multipolar group), vs a CRT-d via bipolar LV pacing (n 96, bipolar group). These patients were followed by clinical, and instrumental assessment, and telemetric device control at follow up. In this study we evaluated, in a population of failing heart T2DM patients, cardiac deaths, all cause deaths, arrhythmic events, CRT-d responders rate, hospitalizations for HF worsening, phrenic nerve stimulation (PNS), and LV catheter dislodgment events (and re-intervention for LV catheter re-positioning), comparing multipolar CRT-d vs bipolar CRT-d group of patients at follow up.

**Results:**

At follow up there was a statistical significant difference about atrial arrhythmic events [7 (7%) vs 16 (16.7%), p value 0.019], hospitalizations for HF worsening [15 (15.2% vs 24 (25%), p value 0.046], LV catheter dislodgments [1 (1%) vs 9 (9.4%), p value 0018], PNS [5 (5%) vs 18 (18.7%), p value 0.007], and LV re-positioning [1 (1%) vs 9 (9.4%), p value 0.018], comparing multipolar CRT-d vs bipolar CRT-d group of patients. Multipolar pacing was an independent predictor of all these events.

**Conclusions:**

CRT-d pacing via multipolar LV lead vs bipolar LV lead may reduce arrhythmic burden, hospitalization rate, PNS, LV catheters dislodgments, and re-interventions in T2DM failing heart patients.

*Clinical trial number* NCT03095196

## Background

Type 2 diabetes mellitus (T2DM) is a worldwide increasing disease [1]. T2DM may impact on heart functions [2], leading to cardiovascular disease [3]. An evident correlation exists between T2DM, and heart failure (HF) disease [4]. In fact, T2DM may condition HF disease progression, its clinical stage, and the response to therapeutic treatments [4]. In HF patients, cardiac resynchronization therapy with a defibrillator (CRT-d) is a well-established treatment to improve symptoms, quality of life, New York Heart Association (NYHA) class, and clinical outcomes [5]. On other hand, the T2DM clinical stage, the glucose homeostasis, the insulin therapy, and the ageing may affect CRT-d response, and the related clinical outcomes [6–12]. In last decades, the advancement of CRT-d technology worked to reduce implant complications, as phrenic nerve stimulation (PNS), and left ventricle (LV) leads dislodgments [13], and to improve clinical outcomes in CRT-d patients [14]. In this setting, CRT-d pacing via a multipolar LV lead may represent one of these technological advancements. In fact, the stable and continuous CRT-d pacing by multipolar LV lead looked to condition the clinical prognosis, and CRT-d responders rate in failing heart patients [14]. At our knowledge, there are not studies investigating these effects in a population of T2DM failing heart subjects. Moreover, our study hypothesis was that, in T2DM failing heart patients multipolar LV pacing may lead to a reduction of PNS episodes, LV leads dislodgments, and interventions to re-positioning LV leads as compared to bipolar LV pacing. These beneficial effects may be associated to a significant reduction of arrhythmic events, hospitalizations for HF worsening, cardiac deaths, and all cause deaths in T2DM patients. Therefore, the aim of the study was to investigate these effects in a population of T2DM failing heart patients randomly treated by CRT-d via multipolar LV lead vs bipolar LV lead. In T2DM failing heart patients, CRT-d via multipolar LV lead vs bipolar LV lead may induce an amelioration of the HF clinical status, and of the CRT-d responders rate.

## Methods

**Fig. 1:**
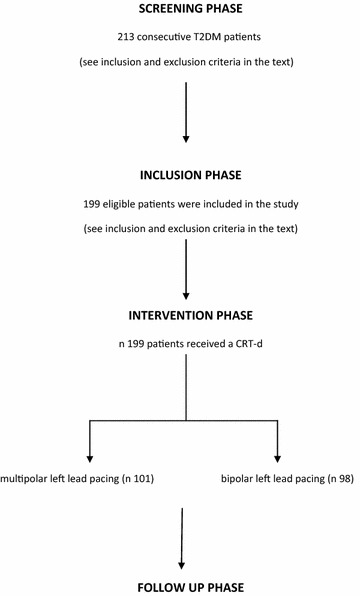
In this figure study flow chart representation. The study was conducted by the following phases: screening phase, inclusion phase, intervention phase, follow up phase. In the screening phase, 213 consecutive T2DM patients [with chronic heart failure lasting for at least 3 months, New York Heart Association (NYHA) functional class II or III, left bundle brunch block, severe left ventricle ejection fraction reduction (LVEF < 35%)], and an indication for cardiac resynchronization therapy with a defibrillator (CRT-d) treatment, were screened to be included in the study (see the inclusion and exclusion criteria in the text). In the inclusion phase 199 patients of this screened population were identified, and included for participation in this study (see inclusion and exclusion criteria in the text). This phase was followed by intervention phase, in that 199 patients received a CRT-d device implant. The CRT-d implant was randomly performed by multipolar (n 101 patients) vs bipolar (n 98 patients) left ventricle pacing lead. After the CRT-d treatment these patients were ambulatory monitored by clinical and instrumental assessment as described in the text during follow up phase. Nine nine patients in multipolar CRT group vs 96 patients in the bipolar CRT group completed the follow up (two patients have refused to participate in the study, one have refused to receive a CRT-d, and one have been lost at follow up)

From September 2012 to September 2015 we conducted a multicenter, prospective, randomized study at University of Campania Luigi Vanvitelli, Italy, Catholic University of Sacred Heart, Campobasso, Italy, and John Paul II Research and Care Foundation, Campobasso, Italy. We screened 213 consecutive T2DM patients (*screening phase*) with stable chronic heart failure, New York Heart Association (NYHA) functional class II or III, left bundle branch block, severe left ventricle ejection fraction reduction (LVEF < 35%), stable sinus rhythm, candidates to receive a CRT-d treatment according to the international guidelines [15, 16] (Fig. 1). Exclusion criteria were as follows: age <18 or >75 years, ejection fraction >35%, previous implantable cardioverter defibrillator (ICD), CRT-d and/or pacemaker implant, absence of informed patient consent, and any condition that would make survival for 1 year unlikely. One hundred and ninety-nine eligible patients were included in the study, and received a CRT-d treatment, and a traditional CRT-d ambulatory monitoring (*inclusion phase*) (Fig. 1). The CRT-d has randomly undergone via multipolar LV lead pacing (n 101) vs bipolar LV lead pacing (n 98) (*intervention phase*) (Fig. 1). We used a computer programming code for treatment randomization. Study population was then divided in multipolar CRT-d group (multipolar LV lead pacing), vs bipolar CRT-d group (bipolar LV lead pacing). Before interventions, baseline laboratory studies, including HbA1c, lipid panel, and fibrinogen, were determined. In this population two patients have refused to participate in the study, one have refused to receive a CRT-d, and one have been lost at follow up. Therefore, 99 patients in multipolar CRT group vs 96 patients in the bipolar CRT group completed the 12 months follow up (*follow up phase*) (Fig. 1). Responders patients to a CRT-d treatment were defined by evidence of LV reverse remodeling, 6 min-walk improvement and Minnesota living with heart failure scale improvement as previous described [15, 16]. Enrolled patients were followed by clinical, instrumental assessment, and device telemetric control (at implant, 10 days, 6th, and 12th months after discharge). During these visits, and device interrogations, we reported lead functionality parameters, and arrhythmic events in CRT-d recipients, PNS episodes, and subsequently CRT-d effect in terms of clinical outcomes, as CRT response entity, and clinical events. The study was conducted in accordance with the Declaration of Helsinki. The Ethics Committees of all participating institutions approved the protocol. All patients were informed about the study nature, and gave their written informed, and signed consent to participate in the study.

### Intervention phase

#### CRT-d implant procedure, and LV pacing leads positioning

Experienced electrophysiologists in CRT implantation performed the three CRT leads positioning in cardiac chambers, and then connected to the CRT-d generator. All CRT-d implant procedures were standardized. Right atrial catheters were all placed in right atrial appendage, and right ventricular catheters in right ventricle apex, as indicated by antero-posterior, right anterior, and left anterior oblique views projections at radioscopic imaging. LV epicardial catheters were placed by percutaneous coronary sinus catheterization in a lateral and/or posterior–lateral target vessel [15, 16]. We randomly chose a multipolar and/or a bipolar LV pacing lead, as described before in the text. After reaching the target left epicardium vessel, we determined the final LV lead position and pacing configuration, by acceptability of pacing thresholds, absence of diaphragmatic stimulation, and anatomic position (chosen position in the target vessel). The final position of the LV pacing lead was assessed with cine fluoroscopy. Implantation duration was defined as the time between skin incision until suture. We used bipolar LV pacing leads (St Jude Medical, Sylmar, CA, USA; Medtronic, Minneapolis, MN, USA), and quadripolar LV pacing leads (Quartet^®^ model 1458Q and Promote Q^®^, St Jude Medical, Sylmar, CA, USA; Attain Performa^®^model, Medtronic, Minneapolis, MN, USA), over-the-wire, steroid eluting with a in-line connector. LV pacing leads were connected to an appropriate bipolar CRT-D device (CRT-d device, St Jude Medical, Sylmar, CA, USA; Medtronic, Minneapolis, MN, USA), and/or to a quadripolar CRT-d device (Quadra Assura CRT-d device, St Jude Medical, Sylmar, CA, USA; Viva^®^ Quad XT and Viva^®^ Quad S cardiac CRT-d, Minneapolis, MN, USA).

### Follow up phase

#### Lead functionality parameters

**Fig. 2:**
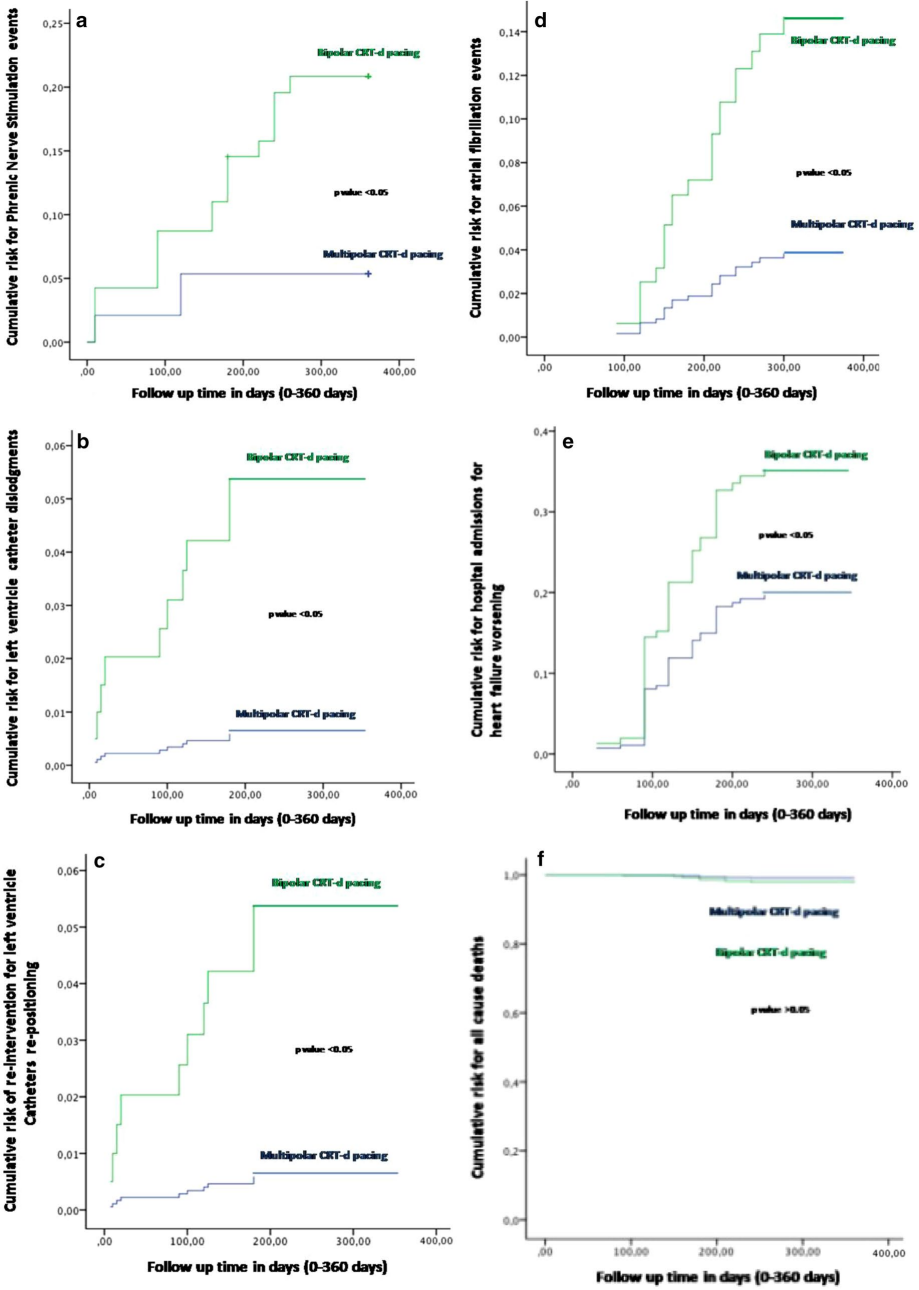
In this figure the representation of cumulative survival events free curves for study endpoints, by Cox regression analysis curves. The figure is structured in seven parts, as **a**–**f**, Fig. 3. In *green color* the bipolar group, in *blue color* the multipolar group for each figure part. The symbol *asterisk* was marking a statistical significant event, as indicated by a p value <0.05. In the part **a** of the figure, the curve representation of Phrenic Nerve stimulation events as “cumulative risk for Phrenic Nerve stimulation” (on *y axis*) during 360 days follow up (on *x axis*) comparing multipolar vs bipolar group. In the part **b** of the figure, the curve representation of catheter dislocation events as “cumulative risk for catheter dislodgement events” (on *y axis*) during 360 days follow up (on *x axis*) comparing multipolar vs bipolar group. In the part **c** of the figure, the curve representation of re-interventions for left ventricle lead re-positioning after dislodgment as “cumulative risk for re-interventions for left ventricle catheter re-positioning” (on *y axis*) during 360 days follow up (on *x axis*) comparing multipolar vs bipolar group. In the part **d** of the figure, the curve representation of hospital admission events as “cumulative risk for hospital admissions events” (on *y axis*) during 360 days follow up (on *x axis*) comparing multipolar vs bipolar group. In the part **e** of the figure, the curve representation of atrial fibrillation events as “cumulative risk for atrial fibrillation events” (on *y axis*) during 360 days follow up (on *x axis*) comparing multipolar vs bipolar group. In the part **f** of the curve, the representation of all cause of deaths events as “cumulative risk for all cause of deaths events” (on y *axis*) during 360 days follow up (on *x axis*) comparing multipolar vs bipolar group

**Fig. 3:**
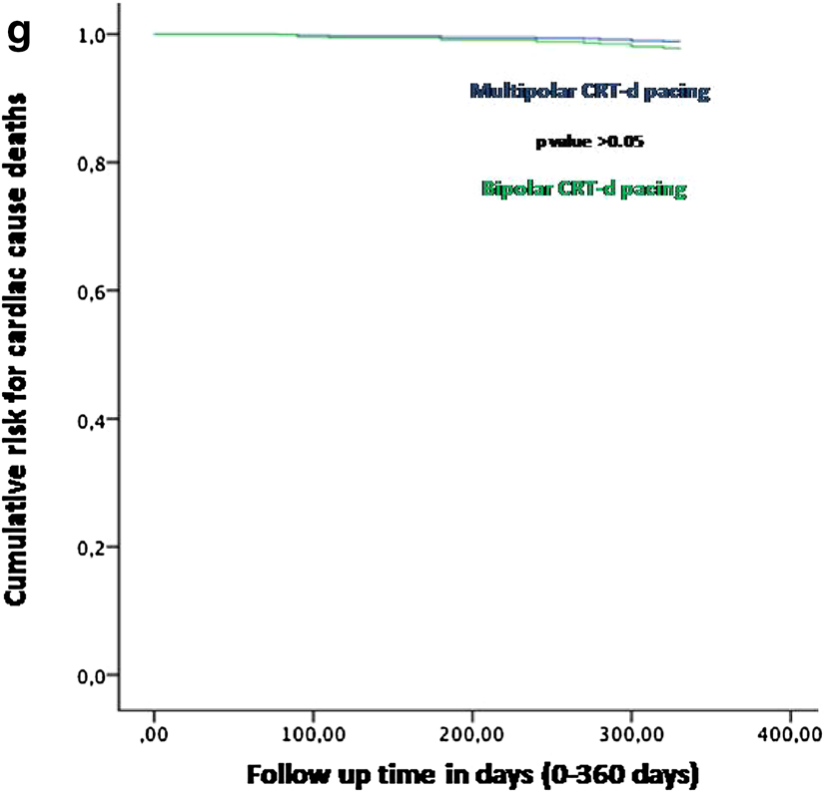
In this figure the representation of cumulative survival events free curves for study endpoints, by Cox regression analysis curves. In the part of the curve, the representation of cardiac deaths events as “cumulative risk for cardiac deaths events” (on *y axis*) during 360 days follow up (on *x axis*) comparing multipolar vs bipolar group

Right atrium, right ventricle, and left ventricle leads functionality parameters (sensing, impedance, and pacing thresholds) were measured as reported, and indicated by international guidelines [15, 16]. These parameters were P, and R-wave amplitude values (sensing thresholds), lead impedances values (impedance thresholds), and lead pacing outputs values (pacing thresholds) (Figs. 2, 3). We monitored, measured, and reported these three parameters according to author’s suggestions [17]. The sensing thresholds values, defined as P wave and R wave sensing amplitude, were obtained from the intra-cardiac electrograms records, measured using a sensing configuration [18, 19]. The pacing thresholds, and impedance thresholds values, were measured using pacing catheter configurations [18, 19]. To measure intra thoracic impedance (Ohm), and pacing thresholds (Volt for ms), we focused on the right ventricle (RV) coil electrode to device case pathway configuration, and on the left ventricle (LV) tip to LV ring configuration [18, 19].

#### Arrhythmic events in CRT-d recipients

In CRT-d patients we monitored and reported atrial fibrillation (AF), ventricular tachycardia (VT), and ventricular fibrillation (VF) episodes, and implantable cardioverter defibrillator (ICD) shocks. AF was defined as paroxysmal, and/or not paroxysmal according to authors’ suggestions [20]. VT was defined as arrhythmia originating from ventricular chambers, sustained and/or not sustained by arrhythmic event duration [21].VF was defined as a fibrillating arrhythmia originating from ventricular chambers, and associated to hemodynamic instability, and cardiac arrest [21]. ICD shocks were defined as high energy interventions by CRT-d device to restore sinus rhythm during at risk of life sustained VT and or VF events [15, 16].

#### Phrenic nerve stimulation events

PNS and left ventricle pacing leads threshold were measured by a standard protocol at CRT-d implant, and during all follow up durations by CRT-d devices interrogations. We evaluated these parameters according to authors’ suggestions, and previous experiences [22]. We tested PNS thresholds during respiratory phases, and supine, left lateral, right lateral, sitting, and standing body position, in each follow up phase, and for every patient. We accepted the pacing configuration (multipolar and bipolar CRT-d pacing) testing the absence of PNS at pacing output of 7.5 V, with a measured pacing threshold less than or equal to 2.5 V. In case of PNS diagnosis during follow up, by patients symptoms assessment, devices interrogations, and physician ambulatory diagnosis (clinical and instrumental PNS diagnosis), we reached the best left ventricle lead pacing configuration to solve PNS. This test was repeated in both clinical group (multipolar vs bipolar LV pacing lead) of CRT-d patients. In this way we reached the final biventricular pacing configuration by LV pacing lead configuration at best pacing thresholds, and in absence of PNS, and after evaluating the effects of different configurations on hemodynamic parameters, as indicated by authors [22].

#### LV leads dislodgments

LV catheter dislodgments resulted by the movement of the catheter into and out of the coronary sinus implantation vessel site, and then causing a change in the catheter tip location [22]. LV catheter dislodgments event was diagnosed by patients clinical symptoms, hospital admissions schedules, hospital discharge schedules, and during medical interrogation at follow up visits. It resulted in altered sensing, pacing, and impedance thresholds of LV lead at device interrogations during follow up. LV catheter dislodgment was confirmed by radiographic biplane projections assessment [22].

#### CRT-d effect on clinical outcomes (interventions for LV lead re-positioning, CRT responders rate, and clinical events)

*CRT*-*d responders rate* was evaluated by periodic clinical examination, and echocardiography assessment [15, 16]. *Interventions for LV lead re*-*positioning* were defined as interventions done after the first CRT-d implant [22], and were evaluated by hospital admissions schedules, hospital discharge schedules, and during medical interrogation at follow up visits. These interventions were performed in case of LV catheter dislodgments. As other clinical outcomes, hospitalization rate was reported during telephonic interviews, by hospital admissions schedules, hospital discharge schedules, and during medical interrogation at follow up visits. *Cardiac deaths, all cause of deaths, and stroke events* were evaluated during office follow up visits 10 days after clinical discharge, and after 6th and 12th months by the treating physician, by telephonic interview, hospital admission, and discharge schedules. At each clinical follow-up, right atrial, right ventricular, and left ventricular leads functionality, *atrial, and ventricular arrhythmias, ICD shocks*, and biventricular pacing percentage, were evaluated and reported for each patient. NYHA classification was re-assessed, and patients graded their overall condition as unchanged or slightly, moderately, or markedly worsened, or improved since randomization by global self-assessment [23]. All patients were instructed to report about devices alarms, loss of lead capture, *phrenic nerve stimulation*, and arrhythmias. All patients were instructed regularly to assess body weight, occurrence of dyspnea, and any clinical symptom. At each visit patients were asked whether medical events or symptoms suggestive of cardiac arrhythmias occurred, and an ECG, and an ECG Holter monitoring, were both performed to detect the presence of asymptomatic arrhythmias. Clinical evaluations included physical examination, vital signs, and review of adverse events. A fasting blood (at least 12 h from last meal) was performed for biochemical peripheral blood assay evaluation at every visit.

### Study endpoints

As *primary endpoints* we monitored CRT-d effect in multipolar CRT-d patients vs bipolar CRT-d patients in terms of PNS episodes, LV leads dislodgments, interventions to re-position LV leads, hospitalization rate for HF worsening, cardiac deaths, and all cause deaths. As *secondary endpoints* we monitored CRT-d responders rate, AF events, VT events, VF events, ICD shocks, and strokes in both groups (multipolar CRT-d vs bipolar CRT-d patients).

### Statistical methods

A qualified statistician analyzed all collected data. The patients were divided before in multipolar CRT-d group vs bipolar CRT-d group, and during follow up visits, and controls in CRT-d responders vs CRT-d non-responders. We postulated that, the number of patients with alterations in primary and secondary endpoints was significantly different between multipolar CRT-d patients vs bipolar CRT-d patients. Safety analyses were performed on data from all enrolled patients. Continuous variables were expressed as means and standard deviations, and were tested by two-tailed Student t test for paired or unpaired data, as appropriate, or by one-way analysis of variance (ANOVA) for more than two independent groups of data. The categorical variables were compared by Chi square or Fisher exact test where appropriate. Survival analysis was performed with the use of the Kaplan–Meier method. Predictors of the study endpoints were evaluated by using Cox regression models. A univariate analysis was conducted to examine the association between single principal clinic, echocardiographic, electrocardiographic characteristics, and multipolar LV pacing, and 12 months study outcomes, as catheter dislodgments, PNS events, re-intervention for catheter dislodgments, hospital admissions for heart failure worsening, and AF events. All variables with p value of less than 0.2 in the univariate analysis were subsequently entered into a multivariate model. In the multivariate model, variables were separately selected and a p value of less than 0.05 was considered significant. For all independent predictors, 95% confidence intervals were calculated. Statistical significance was considered for a p value of less than 0.05. The statistical analysis was performed using the SPSS software package for Windows 17.0 (SPSS Inc., Chicago Illinois).

## Results

One hundred and ninety-five T2DM failing heart patients treated by a CRT-d completed the study follow up, 99 multipolar vs 95 bipolar CRT-d patients (Fig. 1). Mean population age was 67.4 ± 6.5 years in overall population, 68.1 ± 6.6 vs 66.9 ± 6.4 years comparing multipolar vs bipolar CRT-d patients (p value >0.05) (Table 1). One hundred and forty-four patients (74%) in overall population, and 71 (72%) vs 73 (76%) patients comparing multipolar vs bipolar CRT-d patients were males (p value >0.05) (Table 1). Other clinical characteristics, echocardiographic parameters, and drug therapy at enrolment were similar, and balanced between two groups of patients (Table 1). Not differently from these results, we reported similar procedural data as skin to skin time (183 ± 113 vs 179 ± 116 min, p value >0.05), fluoro time (17 ± 4.7 vs 18 ± 5.6 min, p value >0.05), and CS cannulation time (14 ± 5.4 vs 15 ± 4.7 min, p value >0.05), and CRT-d leads functionality parameters (left ventricle, right atrial, and right ventricular leads) comparing multipolar vs bipolar CRT-d patients (Table 2). During follow up, primary and secondary study endpoints were reported in study population, comparing multipolar to bipolar CRT-d patients (Table 3). At follow up, there was a statistical significant difference comparing multipolar to bipolar CRT-d patients, about phrenic nerve stimulation events [5 (5%) vs 18 (18.7%), p value 0.007], catheter displacement events [1 (1%) vs 9 (9.4%), p value 0018], and re-interventions for left leads re-positioning [1 (1%) vs 9 (9.4%), p value 0.018], hospitalizations for heart failure worsening [15 (15.2% vs 24 (25%), p value 0.046], and atrial fibrillation events [7 (7%) vs 16 (16.7%), p value 0.019] (Table 3). At multivariate analysis, quadripolar LV lead vs bipolar LV lead pacing was associated to a reduction of LV catheter dislodgments in a percentage of more than 88% (HR 0.112 [0.014–0.893], 95% CI, p value 0.039), and of 75% for phrenic nerve stimulations events (HR 0.246, [0.088–0.686], 95% CI, p value 0.007) (Table 4). Multipolar LV pacing reduced at more than 88% the cases of re-interventions for LV lead re-positioning (HR 0.112 [0.014–0.893], 95% CI, p value 0.039) (Table 4). Multipolar LV lead vs bipolar LV pacing was associated to a reduction of hospital admissions for heart failure worsening in a percentage more than 48% (HR 0.516, [0.279–0.955], 95% CI, p value 0.035) (Table 4). Multipolar LV lead pacing was associated to a reduction of atrial fibrillation events in a percentage of more than 73% (HR 0.261 [0.086–0.794], 95% CI, p value 0.018) (Table 4). On the contrary, obesity was associated to increased risk to have atrial fibrillation events (HR 1.36, [1.09–1.88], 95% CI, p value 0.02) (Table 4).

**Table 1:** Baseline data of the study cohort (clinical characteristics, echocardiographic parameters, and drug therapy)

Variables	Overall	Multipolar group	Bipolar group	p value
Clinical characteristics				
Patients n	195	99	96	
Age, years	67.4 ± 6.5	68.1 ± 6.6	66.9 ± 6.4	n.s
Male, n (%)	144 (74%)	71 (72%)	73 (76%)	n.s
Smokers (%)	102 (52.3%)	52 (52.5%)	50 (52%)	n.s
Dyslipidemia (%)	102 (52%)	52 (53)	50 (52)	n.s
Obesity	12 (6.1%)	7 (7%)	5 (5.2%)	n.s
Hypertension	136 (69.7%)	71 (71.7%)	65 (67.7%)	n.s
Renal insufficiency (%)	18 (9.2%)	10 (10.1%)	8 (8.3%)	n.s
Ischemic HF (%)	128 (65.6%)	67 (68%)	61 (63.5%)	n.s
Non ischemic HF (idiopathic, hypertensive, or valvular) (%)	67 (34.4%)	32 (32.2%)	35 (36.4%)	n.s
Previous cardiac surgery (%)	35 (18%)	17 (18%)	18 (19%)	n.s
NYHA II (%)	102 (52.3%)	53 (53.5%)	50 (52%)	n.s
NYHA III (%)	93 (47.7%)	49 (49.5%)	44 (45.8%)	n.s
QRS duration	136.4 ± 7.8	137.3 ± 7.4	135.3 ± 8.1	n.s
6MWT	244.1 ± 39.8	239.5 ± 44.7	248.7 ± 33.8	n.s
NT-proBNP (pg/ml)	2307 ± 631	2322 ± 567	2281 ± 723	n.s
Hb1Ac (mmol/mol)	57.8 ± 15.6	57.2 ± 15.3	58.2 ± 16.1	n.s
Echocardiographic parameters				
LVEDv (ml)	198 ± 39	196 ± 31	201 ± 45	n.s
* LVESv* (ml)	137 ± 29	135 ± 23	141 ± 36	n.s
LVEF (%)	27 ± 5	27 ± 5	28 ± 4	n.s
Mitral regurgitation				
+	91 (46.7%)	44 (44.4%)	47(49%)	n.s
++	77 (39.5%)	40 (40.4%)	37 (38.5%)	n.s
+++	19 (9.7%)	9 (9.1%)	10 (10.4%)	n.s
Drug therapy				
ACE-i/ARB	170 (87.2%)	86 (86.8%)	84 (87.5%)	n.s
Beta blockers	137 (70.3%)	70 (71%)	67 (69.8%)	n.s
Diuretics	117 (60%)	58 (58.6%)	59 (61.5%)	n.s
Digoxin	43 (22%)	21 (21.2%)	22 (22.9%)	n.s
Statins	113 (57.9%)	56 (56.6%)	57 (59.4%)	n.s
Insulin	72 (36.9%)	35 (35.3%)	37 (38.5%)	n.s
Oral hypoglycemic drugs	131 (67.2%)	68 (68.7%)	63 (66%)	n.s
Anti platelets drugs	130 (66.7%)	64 (63.6%)	65 (67.7%)	n.s
Dicumarolic anticoagulants	18 (9.2%)	9 (9%)	9 (9.4%)	n.s

In this table clinical characteristics, drug therapy and echocardiographic parameters have been reported, at baseline, of overall population, and then comparing multipolar vs bipolar group of patients. Statistical analysis has been conducted, to compare categorical data, with the exact Pearson’s Χ^2^ test. We considered a two-sided p value of less than 0.05 as statistically significant. The “n.s” was for statistical not significant (p value >0.05)

*ACE-i* angiotensin converting enzyme inhibitor, *ARB* angiotensin receptor II blockers, *COPD* chronic obstructive pulmonary diseases, *Hb1Ac* glycosylated hemoglobin, *y* year, *n* number, *LVEDv* left ventricle end diastolic volume, *LVESv* left ventricle end systolic volume, *LVEF* left ventricle ejection fraction, *6MWT* 6 min walking test, n is for number, *NYHA* New York Hearth Association, *NOACs* new oral anti coagulations drugs, *n.sis* not statistical significant (p value >0.05), *NT-proBNP* N terminal pro B type Natriuretic peptide, in mitral regurgitation the symbol +, ++, +++ indicating low grade (+), mild grade (++), and more than mild (+++) regurgitation grade

**Table 2:** Procedural data of the study cohort

Parameters	Quadripolar group (n 99)	Bipolar group (n 96)	p value
Skin to skin time	183 ± 113	179 ± 116	n.s
Fluoro time	17 ± 4.7	18 ± 5.6	n.s
CS cannulation time	14 ± 5.4	15 ± 4.7	n.s
CS target vessel			
Anterior, n (%)	3 (3)	3 (3)	n.s
Anterolateral, n (%)	13 (13)	11 (12)	n.s
Lateral, n (%)	51 (51)	52 (54)	n.s
Postero-lateral, n (%)	28 (28)	25 (26)	n.s
Posterior, n (%)	4 (4)	5 (5)	n.s
LV lead parameters			
Impedance thresholds	761 ± 182	685 ± 196	n.s
Pacing thresholds	0.5 ± 0.4	0.5 ± 0.3	n.s
Sensing thresholds	14 ± 7.2	13.4 ± 7.3	n.s
RA lead parameters			
Impedance thresholds	438 ± 164	452 ± 133	n.s
Pacing thresholds	0.4 ± 0.2	0.5 ± 0.3	n.s
Sensing thresholds	2.1 ± 1.7	2.3 ± 1.8	n.s
RV lead parameters			
Impedance thresholds	581 ± 151	577 ± 163	n.s
Shock impedance thresholds	73 ± 15	76 ± 14	n.s
Pacing thresholds	0.5 ± 0.45	0.43 ± 0.35	n.s
Sensing thresholds	19 ± 6	18 ± 5	n.s

In this table we reported procedural data as functionality parameters of devices leads (left ventricle, right atrium, and right ventricle leads), and procedural times at implant (shin to skin time, fluoro time, CS target vessel, and CS cannulation time) comparing multipolar vs bipolar group of patients. Impedance thresholds are expressed in Ohm. Pacing thresholds are expressed in millivolts for milliseconds. Sensing thresholds are expressed in millivolts. Procedural times (skin to skin time, fluoro time, and CS cannulation time) are expressed in minutes. The “n.s” was for statistical not significant (p value >0.05)

*CS* coronary sinus, *LV* left ventricle, *RA* right atrium, *RV* right ventricle

**Table 3:** Clinical events of the study cohort

Clinical events	Overall (n 195)	Quadripolar group (n 99)	Bipolar group (n 96)	p value
CRT-d responders rate (%)	117 (60%)	61 (61.6%)	56 (58%)	0.27
Phrenic nerve stimulation	23 (11.8%)	5 (5%)	18 (18.7%)	0.007*
Catheter displacement	10 (5.1%)	1 (1%)	9 (9.4%)	0.018*
Re-interventions	10 (5.1%)	1 (1%)	9 (9.4%)	0.018*
Hospitalizations for HF worsening	39 (20%)	15 (15.2%)	24 (25%)	0.046*
Stroke	4 (2%)	2 (2%)	2 (2%)	0.62
AF n	21 (10.8%)	7 (7%)	16 (16.7%)	0.019*
VT n	51 (26.1%)	25 (25.2%)	26 (27.1%)	0.5
ICD shocks	27 (13.8%)	13 (13.1%)	14 (14.6%)	0.51
Cardiac deaths	9 (5.6%)	4 (4%)	5 (5.2%)	0.43
All cause deaths	12 (6.2%)	5 (5%)	7 (7.3%)	0.33
Stroke	5 (2.6%)	2 (2%)	3 (3.1%)	0.36

In this table are reported clinical events after the CRT-d implant in quadripolar vs bipolar group

*AF* atrial fibrillation, *CRT-d* cardiac resynchronization therapy with a defibrillator, *HF* heart failure, *ICD* internal cardioverter defibrillator, *VT* ventricular tachycardia

The symbol * was marking a statistical significant value, as p value <0.05

**Table 4:** Univariate and multivariate analysis of predictive factors of the study cohort outcomes

	HR	Univariate (95 % CI)	p value	HR	Multivariate (95 % CI)	p value
A. Catheter dislodgments						
Age	0.902	[0.807–1.008]	0.068	0.866	[0.741–1.013]	0.072
Obesity	1.718	[0.218–3.568]	0.608			
Renal dysfunction	1.073	[0.136–4.873]	0.946			
NYHA 3	0.417	[0.108–1.612]	0.205			
QRS duration	0.962	[0.872–1.061]	0.437			
LVEF	1.072	[0.928–1.237]	0.345			
Quadripolar LV	5.278	[1.175–7.32]	0.035*	0.112	[0.014–0.893]	0.039*
B. Phrenic nerve stimulation						
Age	1.024	[0.962–1.089]	0.458			
Obesity	2.604	[0.773–8.767]	0.122	0.658	[0.144–3.01]	0.59
Renal dysfunction	0.043	[0.02–1.79]	0.306			
NYHA 3	2.94	[2.6–4.9]	0.001*	0.97	[0.022–0.423]	0.2
QRS duration	1.019	[0.975–1.065]	0.405			
LVEF	1.012	[0.93–1.101]	0.79			
Quadripolar LV	3.783	[1.404–10.191]	0.008*	0.246	[0.088–0.686]	0.007*
C. Re-intervention for catheter dislodgments						
Age	0.902	[0.807–1.008]	0.068	0.866	[0.741–1.013]	0.072
Obesity	1.718	[0.218–3.568]				
Renal dysfunction	1.073	[0.136–4.873]				
NYHA 3	0.417	[0.108–1.612]				
QRS duration	0.962	[0.872–1.061]				
LVEF	1.072	[0.928–1.237]				
Quadripolar LV	5.278	[1.175–7.32]	0.035*	0.112	[0.014–0.893]	0.039*
D. Hospital admission for heart failure worsening						
Age	0.823	[0.961–1.051]	0.823			
Obesity	1.062	[0.33–3.419]	0.92			
Renal dysfunction	0.898	[0.322–2.501]	0.836			
NYHA 3	1.667	[0.926–3.01]	0.089	0.624	[0.326–1.193]	0.154
QRS duration	1.019	[0.983–1.056]	0.3	1.012	[0.973–1.052]	0.559
LVEF	0.98	[0.926–1.038]				
Quadripolar LV	1.683	[1.04–3.03]	0.05	0.516	[0.279–0. 955]	0.035*
E. Atrial fibrillation events						
Age	1.031	[0.968–1.099]	0.343			
Obesity	5.571	[2.038–15.232]	0.001*	1.36	[1.09–1.88]	0.02*
Renal dysfunction	0.992	[0.231–4.257]	0.991			
NYHA 3	2.58	[1.001–6.649]	0.05*	0.557	[0.195–1.591]	0.274
QRS duration	1.018	[0.971–1.067]	0.457			
LVEF	1.043	[0.95–1.146]	0.375			
Quadripolar LV	3.29	[1.205–8.982]	0.02*	0.261	[0.086–0.794]	0.018*
F. All cause deaths						
Age	1.138	[1.04–1.246]	0.05*	1.149	[0.89–1.288]	0.058
Obesity	2.5	[0.9–2.91]	0.986			
Renal dysfunction	0.88	[0.108–7.193]	0.905			
NYHA 3	1.471	[0.276–7.759]	0.649			
QRS duration	1.051	[0.984–1.123]	0.141			
LVEF	1.069	[0.914–1.249]	0.404			
Quadripolar LV	0.415	[0.104–1.659]	0.214			
G. Cardiac deaths						
Age	1.3	[1.185–1.426]	0.01*	1.332	[0.858–1.532]	0.056
Obesity	0.7	[0.2–2.896]	0.168			
Renal dysfunction	1.676	[0.2–14.02]	0.633			
NYHA 3	0.386	[0.069–2.164]	0.279			
QRS duration	0.932	[0.806–1.078]	0.342			
LVEF	0.912	[0.815–1.044]	0.201			
Quadripolar LV	0.521	[0.154–1.758]	0.293			

In this table the representation of study outcomes, as catheter dislodgments (A), phrenic nerve stimulation events (B), re-intervention for catheter dislodgments (C), hospital admissions for heart failure worsening (D), atrial fibrillation events (E), all cause deaths (F), and cardiac deaths (G), and multivariate predictive factors

We have used for statistical analysis, a 95% interval of confidence (IC), and a significant statistical p value, p < 0.05. The symbol * was marking factor with a p value <0.05. To test the final statistical used model, we have performed the Hosmer and Lemeshow test, with a χ² = 2.775, and a p value <0.05

*NYHA 3* New York Heart Association third class, *LVEF* left ventricle ejection fraction, *LV* left ventricle

## Discussion

In our study the multipolar CRT-d pacing was found to be superior to the bipolar CRT-d pacing respect to the reduction of PNS events, LV leads dislodgment, re-interventions for LV leads dislodgments, hospitalizations for HF worsening, and AF events (Figs. 2, 3; Table 3). The analysis of the others study endpoints did not show differences between the two study groups with regard to cardiac deaths, all cause deaths, strokes, VT events, and ICDs shocks, and CRT-d responders rate (Table 3). In literature less has been reported about the effect of diabetes mellitus on the risks of arrhythmias, and ICDs therapies [24]. Moreover, although authors reported a significant excess of cardiac hospitalizations and mortality in the diabetic population, this higher risk is not related to arrhythmias and/or to a difference in the rate of ICD treatments [24]. We reported a statistical significant reduction of *PNS events* in multipolar group vs bipolar group [n 5 (%) vs n 18 (18.7%), p value 0.007] (Table 3; Fig. 2a). PNS is a challenging, and a relevant problem in failing heart patients treated by CRT-d [25–27], reported in a percentage of 7–14% patients treated by bipolar LV leads, and associated to the LV lead location [14, 28]. In fact, PNS is more common with the LV lead in the mid-apical, posterior and lateral sites, and less common with the LV lead in the anterior or basal site [14, 28]. Multipolar CRT-d pacing may reduce PNS. Physicians may reach and pace the target coronary vessel, and the target LV wall segment, by a LV pacing from electrodes more far from the tip of the lead, that may be more anatomically closed to phrenic nerve course [25–27]. In our study multipolar lead LV pacing was associated to a reduction of *LV leads displacements*, and subsequently of *re*-*interventions* to re-position LV leads [n 1 (1%) vs n 9 (9.4%), p value 0.018], as compared to bipolar LV pacing (Table 3; Fig. 2b, c). In the multivariate analysis, multipolar LV lead pacing was associated to a reduction of *LV leads displacements*, and subsequently of *re*-*interventions* to re-position LV lead, in a percentage of more than 88% (HR 0.112 [0.014–0.893], 95% CI, p value 0.039) (Table 4; Fig. 2b, c). The ability to perform a CRT-d implant may be related to the possibility to cannulate CS, and to reach the target CS vessel [15, 16]. Sometimes, the anatomic position of coronary sinus vessel, the angle of origin of the vessel, the vessel size, and others variables may render difficult to select the target vessel, and it may consequently affect the stability of the implanted LV lead [15, 16]. Other times, the best anatomical, and the consequent more stable position in the target vessel may be related to the position of the tip of LV lead more close to the course of the phrenic nerve, as discussed before, and/or more close to LV segments conditioning worse sensing and pacing thresholds [25–27]. Therefore, a more stable vessel position may sometimes condition the sensing and pacing LV thresholds and programming, while on the contrary the best sensing and pacing LV configuration may not result in the more stable position in the target vessel [25–27]. This may lead to re-positioning of LV lead, in a more proximal position in the target vessel, and/or in a different cardiac vein [15, 16]. These events may be more common seen in bipolar LV leads as compared to multipolar LV leads, by the position of sensing, and pacing couple of poles in the tip of LV bipolar catheter. This may also condition catheter dislodgments after the implant [15, 16, 25–30]. Both these conditions may lead to dislodgments, replacements, and re-interventions of LV leads. Multipolar LV pacing leads may offer a greater variety of sensing and pacing configurations from different sites within a coronary vein, as compared to bipolar pacing [30]. Therefore, this may reduce LV dislodgments, and re-interventions, allowing optimal LV lead placement to maximize biventricular pacing at long term follow up [29, 30]. Moreover, we may prefer the more stable anatomic position in the target vessel, without replacing more proximally the LV lead, and/or finding others veins to reach the best sensing and pacing thresholds and programming [29, 30]. These results were similarly addressed by authors in the overall population of failing heart patients treated by multipolar CRT-d pacing [27]. We may speculate that, the placement of multipolar LV lead in a target LV epicardium vessel, because its anatomical stability, and the possibility to program multiple sensing and pacing thresholds, may result in a stable and continuous CRT-d pacing [26]. Moreover, this may consequently impact on *AF events, and hospitalizations for HF worsening* (Table 3; Fig. 2d, e). In fact, we found that multipolar pacing vs bipolar pacing may lead to a significant reduction of AF events [n 7 (7%) vs 16 (16.7%), p value 0.019]. Intriguingly, the use of multipolar LV pacing during CRT-d implant may be predictive of lower AF events at follow up (HR 0.261 [0.086–0.794], 95% CI, p value 0.018) (Figs. 2, 3; Table 3). This effect CRT-d induced has been discussed by different authors. In the CARE-HF study authors reported that, in sinus rhythm patients the global incidence of AF was similar in medical therapy alone as compared to CRT [31]. On the contrary, others authors reported that CRT-d pacing might be associated with shorter duration of AF events [32]. Therefore, the stable and continuous CRT-d pacing may reduce AF burden [31]. Authors supported this hypothesis by the observation of reversal of left atrial remodeling CRT-d induced, and then associated to shorter duration of AF events during CRT-d pacing [32]. Similarly, same authors showed that AF occurrence was associated with failing heart worsening [32]. These data are in line with our study results. In our study we reported that the obesity was an independent predictor of AF events (HR 1.36 [1.09–1.88], 95% CI, p value 0.02). As first, obesity may be associated to a hyper activation of inflammatory tone, and cytokines expression [33], and to hyper activation of the sympathetic tone [34]. The hyper activation of the sympathetic tone may render obese patients more vulnerable to pro-arrhythmic stimuli [34]. These functional alterations may be consequently associated to structural abnormalities, as abnormal visceral fat deposition, which may lead to an augmentation of atrial arrhythmic burden [35]. These alterations may condition AF burden, without impacting on VT burden [36]. On the contrary, the higher stability of multipolar LV lead, and the consequent continuous biventricular pacing in a target segment of LV wall, may reduce AF events. Consequently the obesity did not diminish the clinical benefit of CRT-d to reduce risk for appropriate ICD therapy, as we observed in our study [36].This effect, and the opportunity to program multiple LV pacing configurations, may be induced by multipolar pacing [37]. The multipolar stable, and continuous biventricular pacing, may induce a stabilization effect on cellular membranes, ionic currents, and cardiac cells [37]. This effect, reducing AF episodes PNS, LV lead dislodgments, and re-interventions to re-position LV leads, may be associated to a lowering of *hospitalizations for HF worsening* in T2DM failing heart patients [n 15 (15.2%) vs 24 (25%), p value 0.046], comparing multipolar pacing vs bipolar pacing (Table 4; Fig. 2d). This may be seen as important study result, and a relevant study message. This result may be seen as a strong data supporting our primary study hypothesis. In fact, it may confirm that, in T2DM failing heart patients the multipolar LV pacing is an advancement of CRT-d pacing safe to use, and that it may be associated to a significant reduction of hospitalizations for HF worsening. In fact, we observed that the choice and the utilization of a multipolar LV pacing lead during CRT-d implant may be an independent predictor factor of hospitalizations for HF worsening (HR 0.516 [0.279–0.955], p value 0.035) in T2DM failing heart patients (Table 4; Fig. 2d). CRT-d reduces the risk of heart-failure events in patients with a low ejection fraction and wide QRS complex [38], and this effect has been reported also in diabetic patients [36]. CRT-d is a class 1 indication to reduce hospitalizations for HF worsening in failing heart diabetic patients, and also in failing heart non diabetic patients [15, 16]. Actually, for the first time in literature we may report that, multipolar pacing vs bipolar pacing may significantly reduce hospitalizations for HF worsening in T2DM failing heart patients. Parallely, we may observe that, the choice of multipolar CRT-d pacing may be predictive of hospitalizations lowering for HF worsening in T2DM failing heart patients. In our study, the reduction of HF events induced by multipolar CRT-d pacing, was not associated to a reduction of the mortality (cardiac deaths, and all cause deaths). Similarly, multipolar CRT-d pacing in T2DM patients did not significantly improve the CRT-d responder’s rate. These results have to be seen in the complexity of diabetes, and of its effect on failing heart disease, and on clinical outcomes in CRT-d recipients [11, 12]. All these results, such as the not significant improvement in the percentage of CRT-d responders, may be related to higher percentage of ischemic cardiac disease conditioning HF in T2DM patients, and then impacting on CRT-d response [1–4, 6–12].

### Study limitations

This study had few limitations. As first, we examined a small percentage of T2DM failing heart patients treated by multipolar vs bipolar CRT-d, as compared to overall population. This was due to loss of patients during follow up, and to the low adherence of patients to the study protocol as discussed in results session. Second, this study was conducted at 12 months follow up time, and this short time follow up duration may affect the long term follow up prognosis, and primary and secondary clinical outcomes. Third, we have to report the paucity of clinical characteristics that would provide a more accurate comparison to clinical trial subjects. At last we have not investigated the molecular, and epigenetic aspects induced by T2DM in failing heart patients, and/or modulated by CRT-d. In fact, T2DM by an altered glucose homeostasis may induce electrophysiological changes, which leads to QRS prolongation, decreased conduction velocity and increased arrhythmogenesis, and this may condition clinical prognosis [39]. We mentioned data about cardiac electrophysiology properties by CRT devices interrogations, examined, and collected during routine devices interrogations. We did not report data by continuous devices monitoring systems [40]. This may be limiting for our analysis, because continuous monitoring of CRT-d devices may impact positively on clinical outcomes [40]. We did not perform imaging examinations to support cases of left atrial remodeling observed in study population CRT, and associated to shorter duration of AF events as described by authors [30]. In this study we did not perform animal experiments to test these clinical effect induced by multipolar CRT-d pacing in T2DM failing heart subjects, and we loss data about cardiac cells electrical properties, inflammatory tone, oxidative stress modulation, and sympathetic tone activity in T2DM patients. In this study we did not report data about the epigenetic effect CRT-d induced in T2DM failing heart patients, and this may be a limiting factor of our study analysis [41, 42]. In this study we did not compare multipolar Vs bipolar CRT-d pacing in failing heart diabetic vs non diabetic patients.

## Conclusion

In T2DM failing heart patients multipolar CRT-d pacing may reduce PNS, catheters dislodgments, and re-interventions as compared to bipolar CRT-d pacing. This effect may be associated to reduction of AF events, and hospitalizations for HF worsening. Multipolar CRT-d pacing may offer a relevant hemodynamic advantage over a fixed conventional CRT-d pacing by a single site LV lead in failing heart patients [43, 44]. In our study, we focused on these hemodynamic, and clinical effects induced by multipolar CRT-d pacing in a population of T2DM failing heart patients. In fact, in T2DM failing heart patients multipolar LV pacing vs bipolar LV pacing may lead to a statistical significant reduction of hospital admissions for HF worsening. This study endpoint reached by multipolar CRT-d pacing, was not correlated to a statistical significant improvement of CRT-d responders rate in T2DM failing heart patients. Finally, multipolar CRT-d did not affect cardiac deaths, and all cause deaths in a population of T2DM failing heart patients. A part of this, we may speculate that, multipolar CRT-d pacing may represent a possible way to reduce CRT-d failures, and to improve clinical outcomes in T2DM failing heart patients. In fact, multipolar LV pacing may be predictive, as compared to bipolar pacing, of all these adverse procedure related events, and clinical events. We may speculate that, the lowering of AF events, and hospitalizations for HF worsening, and the predictably of these adverse conditions, may lead us to prefer a multipolar vs bipolar pacing in a population of T2DM failing heart patients. Moreover, we may propose multipolar CRT-d pacing as an innovative, and better treatment in T2DM failing heart patients.

## Abbreviations

AF: atrial fibrillation
CRP: C reactive protein
CRT-d: cardiac resynchronization therapy with a defibrillator
HF: heart failure
ICD: implantable cardioverter defibrillator
LV: left ventricle
LVEF: left ventricle ejection fraction
ms: millisecond
mV: millivolt
NYHA: New York Heart Association
PNS: phrenic nerve stimulation
RV: right ventricular
T2DM: type 2 diabetes mellitus
VF: ventricular fibrillation
VT: ventricular tachycardia
